# Identifying Orbital Angular Momentum of Vectorial Vortices with Pancharatnam Phase and Stokes Parameters

**DOI:** 10.1038/srep11982

**Published:** 2015-07-10

**Authors:** Dengke Zhang, Xue Feng, Kaiyu Cui, Fang Liu, Yidong Huang

**Affiliations:** 1 Department of Electronic Engineering, Tsinghua National Laboratory for Information Science and Technology, Tsinghua University, Beijing 100084, China

## Abstract

In this work, an explicit formula is deduced for identifying the orbital angular moment (OAM) of vectorial vortex with space-variant state of polarization (SOP). Different to scalar vortex, the OAM of vectorial vortex can be attributed to two parts: 1. the azimuthal gradient of Pancharatnam phase; 2. the product between the azimuthal gradient of orientation angle of SOP and relevant solid angle on the Poincaré sphere. With our formula, a geometrical description for OAM of light beams can be achieved under the framework of the traditional Poincaré sphere. Numerical simulations for two types of vectorial vortices have been carried on to confirm our presented formula as well as demonstrate the geometrical description of OAM. Furthermore, this work would pave the way for precise characterization of OAM charge of vectorial vortices.

It is well-known that light carries both linear and angular momenta while the angular momenta (AM) can be divided into spin angular momentum (SAM) and orbital angular momentum (OAM)^1^,^2^,^3^. Under paraxial approximation, it is generally believed that SAM and OAM are associated with polarization and spatial profile of the light fields, respectively^4^. As explicated by Allen *et al.* in 1992^5^, a scalar vortex field with wavefront of 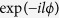 holds discrete OAM of 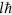 per photon, where 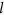 is the topological charge. Thus, for scalar vortices, the topological charge is directly related to the OAM of light beam. However, for vectorial vortex fields, even in the paraxial approximation, only the helical wavefront is not sufficient to characterize OAM just by utilizing topological charge while the state of polarization (SOP) of light field should also be taken into account^6^,^7^. As demonstrated by Wang *et al.* in 2010^8^, besides the azimuthal phase gradient, the OAM also can be generated from the curl of polarization in a vectorial vortex field. Meanwhile, Hasman *et al.* declared that there is a link between OAM and geometric phase induced by space-variant SOP of light fields^9^,^10^,^11^. But so far, the explicit relation between OAM and phase distribution in vectorial vortex fields is still veiled.

In this work, we have deduced that, for vectorial vortex, the OAM can be attributed to two parts. The first is the azimuthal gradient of Pancharatnam phase while the other is the product between the azimuthal gradient of orientation angle of SOP and the related solid angle on the Poincaré sphere. Numerical simulations have been carried on vectorial vertices generated by superposition of two scalar vortex fields and phased array antenna, respectively. Both of them have confirmed our deduced relation. It should be emphasized that our deduced formula of OAM charge is expressed with normal Stokes parameters so that the traditional Poincaré sphere can be utilized to fully characterize both the SAM and OAM. It indicates that geometrical description and characterization of OAM can be achieved by adopting the fundamental Poincaré sphere, which is different to previous reports based on multiple high-order Poincaré spheres^12^,^13^,^14^. On the other hand, as measuring Stokes parameters is a standard measurement of polarization state, it can be expected that such formula could provide an effective and accurate method for identifying the OAM charge, which is very urgent in practical application of OAM beams^15^,^16^,^17^,^18^,^19^,^20^. Meanwhile, because of the explicit expression between OAM and SOP, we believe that this work would provide a new insight of studies on the vectorial vortices, spin-orbit interaction, and such related fields^21^,^22^,^23^,^24^,^25^,^26^.

## Results

### Theoretical description

Under the paraxial approximation, the electric field of a fully polarized vectorial vortex beam with angular frequency 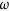 propagating along 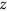 direction in free space can be written as^27^


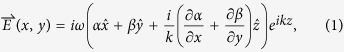


where 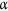 and 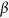 represent the complex amplitude of 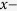 and 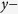 component of electric field, respectively. Obviously, such a vectorial vortex beam has space-variant SOP and its 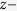 component of angular momentum density can be calculated and divided into spin and orbital parts in cylindrical coordinate system as


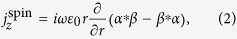



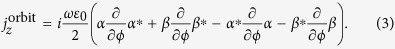


As demonstrated in Ref. 12, an effective tool to describe the SOP of light is the Poincaré sphere with Stokes parameters. Thus, in this work, Stokes parameters and the Poincaré sphere are also introduced to deduce the relation between OAM and SOP. In equations (2) and (3), the complex amplitudes of 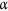 and 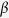 can be written as 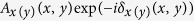, where 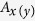 and 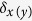 are amplitude and phase (both are real numbers), respectively. Then, the Stokes parameters can be defined as^28^


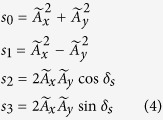


where 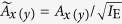 are normalized to the electric intensity of 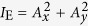 and 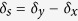 is the phase difference between 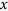 and 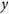 components. Then using 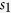, 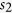, and 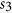 as the sphere’s Cartesian coordinates, the Poincaré sphere is constructed and its corresponding orientation angle 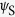 of SOP on the Poincaré sphere can be resolved by


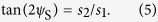


With the ratio of angular momentum to energy that is examined by Allen^29^, the average SAM charge and OAM charge of a vortex beam can be calculated. The SAM charge can be solved by calculating the SAM density with 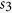, which directly represents the polarization degree^28^. While for OAM charge, there is no explicit connection with Stokes parameters. According to the feature of space-variant SOP in vectorial vortex fields, Pancharatnam phase is adopted to reveal the phase distribution for a vectorial vortex beam as shown in Ref. 11. The reason is that Pancharatnam phase can well describe the phase difference of lights with different SOP while the OAM is a quantity related to the phase distribution of lights. As described in Ref. 30, Pancharatnam phase is defined as 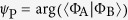 between two different SOP of 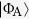 and 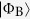. Based on mode expansion theory, any optical beam can be expanded by right and left circularly polarized light, which are written as 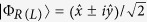. For the same reason, in the paper, the right or the left circularly polarized field is set as a reference field. Then the Pancharatnam phase of the investigated vectorial vortex field 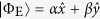 (defined by equation (1)) to the reference field is given by


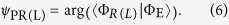


After some derivations (detailed in supplementary information), by applying the orientation angle 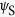 of SOP on the Poincaré sphere and Pancharatnam phase 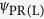 defined by equations (5) and (6), the average OAM charge can be resolved as


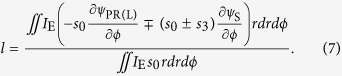


In bracket of numerator of equation (7), the first term is the gradient of spiral spatial phase, which is the topological Pancharatnam charge similar to definition in Ref. 11 and could be understood as the counterpart of topological charge in scalar vortex fields. The second term is related to the variation of SOP in space, which could be analyzed with the Poincaré sphere. To illustrate the physical interpretations and applicable scope of equation (7), in the following section, two cases are demonstrated, where vectorial vertices are generated by superposition of two scalar vortex fields and phased array antenna.

### Superposition of two scalar vortex fields

For general vector beams, such as radially and azimuthally polarized light, the field can be generated according to the following form^31^


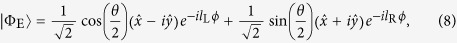


where 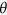 is zenith angle in spherical coordinate (the Poincaré sphere), and the set 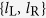 is topological charge of field components with left and right circular polarization respectively. For a fully polarized light (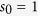), there is a relation of 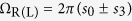. Here 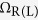 is the solid angle formed by the swept surface area of SOP revolving around the south (north) pole on the Poincaré sphere. Thus, equation (7) can be rewritten as


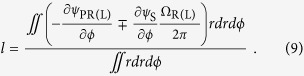


With field expression in equation (8), azimuthal gradients of the Pancharatnam phase and the orientation angle could be analytically expressed as (details are in supplementary information)


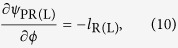



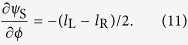


Thus, substituting equations (10) and (11) into equation (9), the OAM charge is obtained as


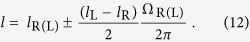


**Figure 1:**
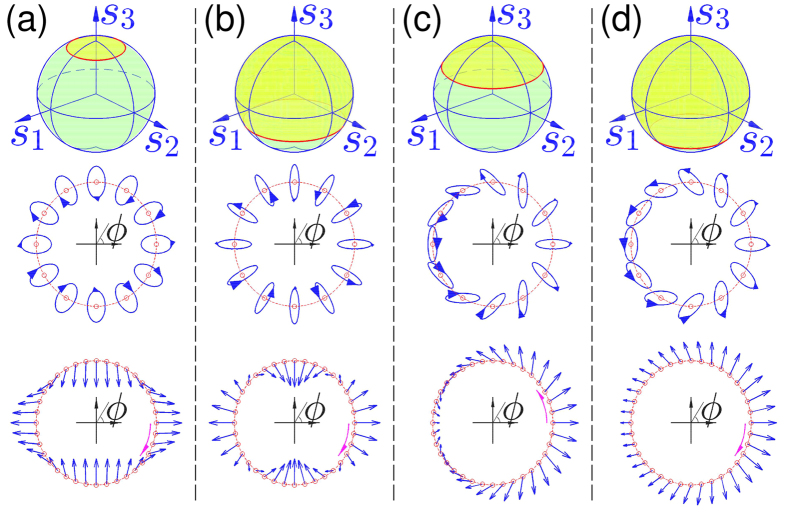
SOP distributions. Four vector beams with azimuthal variant state of polarization (SOP) generated with equation (8) are shown in four row panels, corresponding to (**a–d**). In each panel, different sketches of SOP trace on the Poincaré sphere marked by red line, SOP distribution in space and snap picture of SOP are demonstrated in order. Associated parameters in equation (8) for field generation are (**a**) 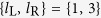, 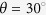, (**b**) 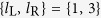, 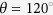, (**c**) 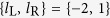, 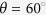, and (**d**) 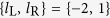, 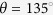, respectively.

In the right side of equation (12), the first term corresponds to topological Pancharatnam charge (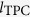), which is referenced to right or right circularly polarized field and just equals to 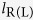 in this case. The second term is the SOP-related charge, which is the product of the azimuthal gradient of orientation angle of SOP and the related solid angle on the Poincaré sphere. For more clarity, some simulations have been carried on four fields generated with equation (8) and the results are shown in Fig. 1.

**Figure 2:**
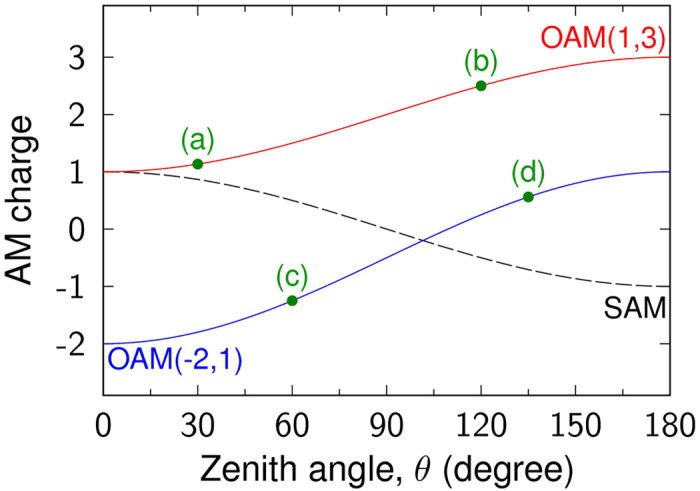
Charges of vector beams generated by superposition of scalar vortices. The calculated OAM charge for the vector beams generated with equation (8) of 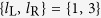 and 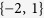 at different zenith angle in spherical coordinate (the Poincaré sphere). Green dots of OAM charges are calculated with our formula, which are corresponding to cases shown in Fig. 1(a–d), respectively. Solid lines are calculated by mode expansion method according to equation (8).

Figure 1(a–d) are the calculated results while the parameters are set as 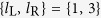 with 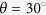 and 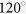 and 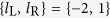 with 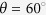 and 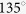. For each row panel, there are three parts in order: SOP trace on the Poincaré sphere marked by red line, SOP distribution in space, and a SOP snap in space. In Fig. 2, calculated results of OAM charges are shown as the green dots, which are calculated by equation (12) for the four cases shown in Fig. 1(a–d). For comparison, the OAM charges are also calculated by mode expansion method according to equation (8) and shown as solid lines in Fig. 2. For the cases shown in Fig. 1(a,b), the left circularly polarized fields (North Pole on the Poincaré sphere) is selected as the reference field and swept surface areas are also shown with yellow zone. While for Fig. 1(c,d), right circularly polarized field (South Pole on the Poincaré sphere) is selected as the reference. In Fig. 2, all the calculated results with our formula are in good agreement with those calculated by mode expansion method. From the results shown in Figs 1 and 2, a clear relation of OAM charge versus Pancharatnam phase, orientation angle of SOP, and the related solid angle on the Poincaré sphere is presented. Furthermore, with our formula, a geometrical description of OAM can be obtained by utilizing a basic Poincaré sphere, as shown in Fig. 1.

### Phased array antenna

**Figure 3:**
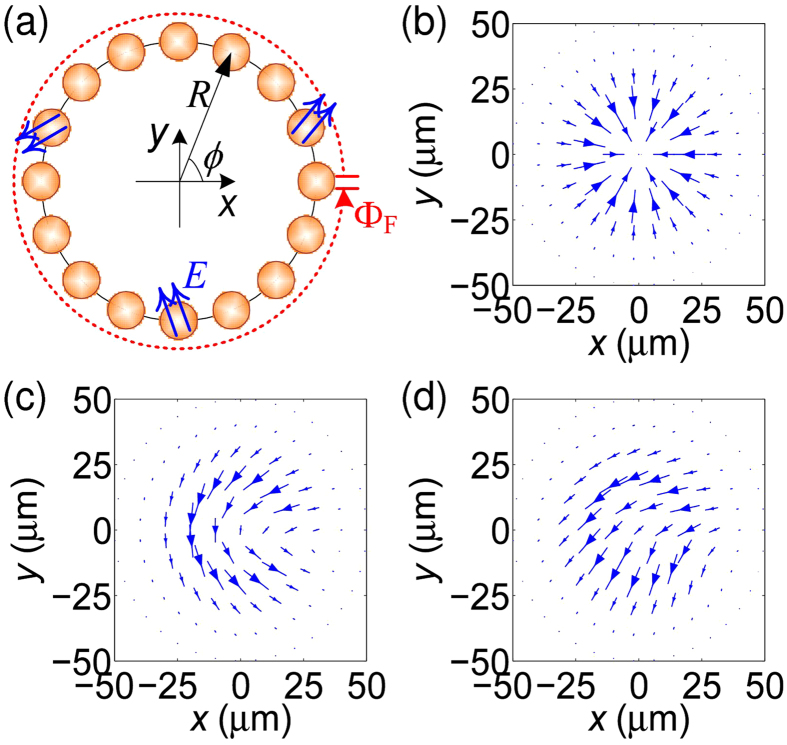
Vectorial vortices generated with PAA. (**a**) Schematic of the considered phased array antenna (PAA), which consists of 16 units. Each unit emits linearly polarized Gauss beam and the polarization direction and initial phase can be set. With PAA, varied vectorial vortex beams can be generated, (**b**) state of polarization (SOP) makes two revolutions at latitude on the Poincaré sphere (radially polarized vectorial beam), (**c**) SOP makes one revolution (L-line vortex), and (**d**) SOP makes half revolution.

Recently, more and more attentions have been focused on the generation of OAM beams with phased array antenna (PAA) in RF, microwave, and lightwave region^32^,^33^,^34^,^35^. To model such process, some simulations are also carried on an annular PAA with antenna unit of linearly polarized Gauss beam as schematically shown in Fig. 3(a). In this case, the optical communication wavelength of 1550 nm is adopted. For each Gauss beam, the waist size is 8 μm and polarization direction is azimuthal-dependent. The unit number is 16 and radius (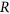) of annular PAA, which is defined by the distance between the PAA center and each unit center as marked in Fig. 3(a), can be adjusted. These parameters ensure that the generated beam satisfies the paraxial approximation, thus Barnett’s method^3^ can be utilized as a reference with the results calculated by equation (7). As demonstrated in Ref. 33, the AM charges of the generated beam can be tuned by varying the phase difference between adjacent units. In our simulation, the adjacent phase differences are uniform and the whole feeding phase of a circle (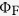) is used to descript the setting phase of PAA. With such a structure, various vortex beams can be generated, such as radially or azimuthally polarized vector beams, L-line vortex beams^36^, and so on.

**Figure 4:**
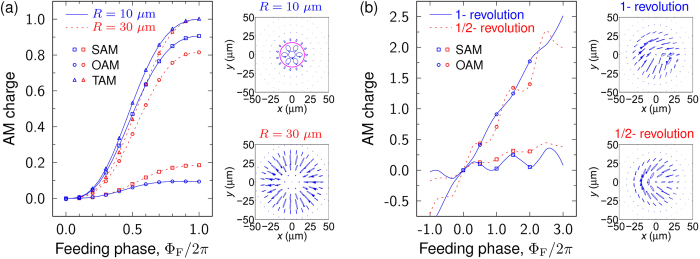
Charges of vectorial vortices generated by PAA. Calculated angular momentum (AM) charges of (**a**) the vortex beam shown in Fig. 3(b) at different feeding phase of two different PAA radius of 10 and 30 μm, and (**b**) the vortex beam shown in Fig. 3(c,d) at different feeding phase of fixed PAA radius of 20 μm. In the figures, lines are calculated by Barnett’s method and symbols are calculated by our formula for OAM and Stoke parameter of 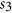 for SAM. Right-side insets also show the corresponding SOP distribution at feeding phase of 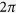.

Figure 3(b) shows a radially polarized vectorial beam, where SOP makes two revolutions at latitude on the Poincaré sphere for a circle in the space. Corresponding AM charges are calculated by both Barnett’s method and our formula, which are shown as lines and dots in Fig. 4(a), respectively. Two cases with different PAA radius of 10 and 30 μm are also considered under the varied feeding phase, both methods give consistent OAM charge. Furthermore, Fig. 3(c,d) display another two types of vortex beam, where SOP makes one and half revolution at latitude on the Poincaré sphere for a circle in the space, respectively. For a fixed PAA radius of 20 μm, the OAM charges at different feeding phase are also calculated and presented in Fig. 4(b), and again, they are also in a very good agreement. These results indicate that the calculations for OAM charge with equation (7) can be applied on not only general vector beams but also complex vortex beams under the paraxial approximation, which can be explained by the principle of superposition with basis beams^37^.

## Discussion

It should be noticed that, for most general vector beam shown in Fig. 3(b), the feeding phase 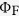 are transferred to both OAM and SAM (see Fig. 4(a)), which is quite different to the scalar vortex beam. For a scalar vortex beam, the feeding phase 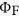 would be fully transferred to OAM. However, for the vectorial vortices, even in the cases of 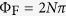 (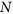
is any integer number), the number of 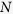 is equals to the total angular momentum (TAM) charge of generated beam, while not the value of OAM charge (recently, a similar report was presented in Ref. 38). The reason is that part of the feeding phase is transferred to SAM in the central zone of vortex as shown with magenta circle in right-side inset of Fig. 4(a) and meanwhile the reduction of solid angle of swept area on the Poincaré sphere would suppress the transformation of OAM from feeding phase. Fortunately, through a carefully designed PAA, the proportion of OAM charge can be varied by reducing power proportion of field around vortex center. For the same reason of partial OAM induced by azimuthal gradient of SOP-related phase, the detection of OAM charge will be different with that for scalar vortex by only detecting phase angle of wavefront. Thus, to detect OAM charge of vectorial vortices, a new method is required. Here, we can expect that such detection can be achieved by traditional measurement of Stokes parameters according to equation (7).

In equation (7), we introduce a reference field to calculate the OAM charge of vortices. In this work, only special reference fields, SOP of right or left circular polarization, were selected. However, it does not mean that a general reference field would induce an incorrect calculation result of OAM charge. To demonstrate it, a series of simulation were carried out to make a contrast, which is explained with more details in supplementary information. Although the selection of reference field does not affect the result of OAM charge, reference field with right or left circular polarization is a normal choice in the measurement of Stokes parameters as well as this choice can also obtain a simple and elegant expression of OAM charge as equation (7).

## Conclusion

In summary, for paraxial vectorial vortex beams propagating in free space, it is deduced that the OAM charge is not only related with the topological Pancharatnam charge but also the SOP-related charge induced by space-variant state of polarization (SOP). Based on such a connection, OAM also can be fully represented by the fundamental Poincaré sphere. And we can expect that the OAM charge can be detected by testing Stokes parameters, which is a standard test of polarization measurement for antennas. Moreover, because of the explicit relation with SOP, we believe that this work would give some new insights for studies on vectorial vortices, spin-orbit interaction, photonic topological insulators, and so on.

## Additional Information

**How to cite this article**: Zhang, D. *et al.* Identifying Orbital Angular Momentum of Vectorial Vortices with Pancharatnam Phase and Stokes Parameters. *Sci. Rep.* **5**, 11982; doi: 10.1038/srep11982 (2015).

## Supplementary Material

Supplementary Information
