# Supplementary material for: Identifying Orbital Angular Momentum of Vectorial Vortices with Pancharatnam Phase and Stokes Parameters
Source: Sci Rep. 2015 Jul 10;5:11982. doi: 10.1038/srep11982 (PMC4498175; doi:10.1038/srep11982)
Supplement: Supplementary Information [file srep11982-s1.pdf]

Supplementary Information

Identifying Orbital Angular Momentum of Vectorial Vortices with Pancharatnam Phase and Stokes Parameters

Dengke Zhang, Xue Feng*, Kaiyu Cui, Fang Liu, and Yidong Huang

Department of Electronic Engineering, Tsinghua National Laboratory for Information Science and Technology, Tsinghua University, Beijing 100084, China.

**Corresponding author: x-feng@tsinghua.edu.cn*

A. Relation of orbital angular momentum, Pancharatnam phase, and Stokes Parameters

Under the paraxial approximation, the electric and magnetic fields of a fully polarized vectorial vortex beam of angular frequency ω propagate along z direction can be written as [S1]

$$\vec{E}(x,y) = i\omega\left(\alpha\hat{x} + \beta\hat{y} + \frac{i}{k}\left(\frac{\partial\alpha}{\partial x} + \frac{\partial\beta}{\partial y}\right)\hat{z}\right)e^{ikz}, \tag{S1a}$$

$$\vec{B}(x,y) = ik\left(-\beta\hat{x} + a\hat{y} + \frac{i}{k}\left(-\frac{\partial\beta}{\partial x} + \frac{\partial\alpha}{\partial y}\right)\hat{z}\right)e^{ikz}, \tag{S1b}$$

where α and β represent the complex amplitude of x and y component of electric field. They can be written as

$$\alpha(x,y) = A_x(x,y)\,\mathrm{e}^{-i\delta_x(x,y)}, \tag{S2a}$$

$$\beta(x,y) = A_y(x,y)\,\mathrm{e}^{-i\delta_y(x,y)}, \tag{S2b}$$

where $A_{x(y)}$ and $\delta_{x(y)}$ are real numbers and represent amplitude and phase, respectively. Thus, Stokes parameters are defined by [S2]

$$\begin{aligned} s_0(x,y) &= \tilde{A}_x^2 + \tilde{A}_y^2 \\ s_1(x,y) &= \tilde{A}_x^2 - \tilde{A}_y^2 \\ s_2(x,y) &= 2\tilde{A}_x\tilde{A}_y\cos\delta_s \\ s_3(x,y) &= 2\tilde{A}_x\tilde{A}_y\sin\delta_s \end{aligned} \tag{S3}$$

where $\tilde{A}_{x(y)} = A_{x(y)}\big/\sqrt{I_\mathrm{E}}$ are normalized electric field components with electric intensity of $I_\mathrm{E} = A_x^2 + A_y^2$, and $\delta_s = \delta_y - \delta_x$ is the phase difference between x and y electric field components. Then using s_1, s_2, and s_3 as the sphere's Cartesian coordinates, the Poincaré sphere is constructed and the corresponding spherical

angles $(2\psi_S, 2\chi_S)$ are resolved by [S2]

$$\tan(2\psi_S) = s_2/s_1, \tag{S4a}$$

$$\sin(2\chi_S) = s_3/s_0. \tag{S4b}$$

The linear momentum density, which is defined as of $\bar{p} = \varepsilon_0 \bar{E} \times \bar{B}$, can be written and divided into transverse and longitudinal components

$$\bar{p}_\perp = i\frac{\omega\varepsilon_0}{2}\left[\left(\alpha\nabla\alpha^* + \beta\nabla\beta^* - \alpha^*\nabla\alpha - \beta^*\nabla\beta\right) + 2\nabla\times\left(\left(\alpha^*\beta - \beta^*\alpha\right)\hat{z}\right)\right], \tag{S5a}$$

$$p_z = \omega k\varepsilon_0\left(|\alpha|^2 + |\beta|^2\right) = \omega k\varepsilon_0 I_E s_0. \tag{S5b}$$

Meanwhile, the energy density of such a beam is

$$w = cp_z = \varepsilon_0\omega^2\left(|\alpha|^2 + |\beta|^2\right) = \varepsilon_0\omega^2 I_E s_0. \tag{S6}$$

Then, the cross product of line momentum density with $\bar{r}$ (radius vector) gives the angular momentum density, and z component of angular momentum density is

$$\begin{aligned} j_z &= \left(\bar{r}\times\bar{p}\right)_z = rp_\phi \\ &= i\frac{\omega\varepsilon_0}{2}\left[\left(\alpha\frac{\partial}{\partial\phi}\alpha^* + \beta\frac{\partial}{\partial\phi}\beta^* - \alpha^*\frac{\partial}{\partial\phi}\alpha - \beta^*\frac{\partial}{\partial\phi}\beta\right) + 2r\frac{\partial}{\partial r}\left(\alpha^*\beta - \beta^*\alpha\right)\right]. \end{aligned} \tag{S7}$$

Further， j_z can be divided into spin and orbital parts as

$$j_z^{\text{spin}} = i\omega\varepsilon_0 r\frac{\partial}{\partial r}\left(\alpha^*\beta - \beta^*\alpha\right) = \omega\varepsilon_0 r\frac{\partial\left(I_E s_3\right)}{\partial r}, \tag{S8a}$$

$$\begin{aligned} j_z^{\text{orbit}} &= i\frac{\omega\varepsilon_0}{2}\left(\alpha\frac{\partial}{\partial\phi}\alpha^* + \beta\frac{\partial}{\partial\phi}\beta^* - \alpha^*\frac{\partial}{\partial\phi}\alpha - \beta^*\frac{\partial}{\partial\phi}\beta\right) \\ &= \omega\varepsilon_0 I_E\left(\tilde{A}_x^2\frac{\partial\delta_x}{\partial\phi} + \tilde{A}_y^2\frac{\partial\delta_y}{\partial\phi}\right). \end{aligned} \tag{S8b}$$

With the ratio of angular momentum over energy that is examined by Allen [S3], the average SAM charge and OAM charge can be calculated as

$$s = \omega\frac{\iint j_z^{\text{spin}} r dr d\phi}{\iint w r dr d\phi} = \frac{\iint I_E s_3 r dr d\phi}{\iint I_E s_0 r dr d\phi}, \tag{S9a}$$

$$l = \omega\frac{\iint j_z^{\text{orbit}} r dr d\phi}{\iint w r dr d\phi} = \frac{\iint I_E\left(\tilde{A}_x^2\frac{\partial\delta_x}{\partial\phi} + \tilde{A}_y^2\frac{\partial\delta_y}{\partial\phi}\right) r dr d\phi}{\iint I_E s_0 r dr d\phi}. \tag{S9b}$$

Then, introducing Pancharatnam phase for two different SOP of $|\Phi_A\rangle$ and $|\Phi_B\rangle$, which is defined by [S4]

$$\psi_P = \arg\left(\langle\Phi_A|\Phi_B\rangle\right). \tag{S10}$$

Here, using right or left circularly polarized fields as reference field, the Pancharatnam Phase of any field $|\Phi_{\mathrm{E}}\rangle = \alpha\hat{x} + \beta\hat{y}$ can be written as

$$\psi_{\mathrm{PR(L)}} = \arg\left(\langle\Phi_{\mathrm{R(L)}}|\Phi_{\mathrm{E}}\rangle\right). \tag{S11}$$

Then we can obtain

$$\tan\psi_{\mathrm{PR(L)}} = \frac{\tilde{A}_y\cos\delta_y \pm \tilde{A}_x\sin\delta_x}{\tilde{A}_y\sin\delta_y \mp \tilde{A}_x\cos\delta_x}. \tag{S12}$$

Further, we can deduce the azimuthal gradient of the Pancharatnam Phase as

$$\frac{\partial\psi_{\mathrm{PR}}}{\partial\phi} = \frac{-\cos\delta_s}{s_0 - s_3}\left(\tilde{A}_x\frac{\partial\tilde{A}_y}{\partial\phi} - \tilde{A}_y\frac{\partial\tilde{A}_x}{\partial\phi}\right) + \frac{s_3}{2(s_0 - s_3)}\left(\frac{\partial\delta_x}{\partial\phi} + \frac{\partial\delta_y}{\partial\phi}\right) - \frac{1}{s_0 - s_3}\left(\tilde{A}_x^2\frac{\partial\delta_x}{\partial\phi} + \tilde{A}_y^2\frac{\partial\delta_y}{\partial\phi}\right) \tag{S13a}$$

$$\frac{\partial\psi_{\mathrm{PL}}}{\partial\phi} = \frac{\cos\delta_s}{s_0 + s_3}\left(\tilde{A}_x\frac{\partial\tilde{A}_y}{\partial\phi} - \tilde{A}_y\frac{\partial\tilde{A}_x}{\partial\phi}\right) - \frac{s_3}{2(s_0 + s_3)}\left(\frac{\partial\delta_x}{\partial\phi} + \frac{\partial\delta_y}{\partial\phi}\right) - \frac{1}{s_0 + s_3}\left(\tilde{A}_x^2\frac{\partial\delta_x}{\partial\phi} + \tilde{A}_y^2\frac{\partial\delta_y}{\partial\phi}\right) \tag{S13b}$$

On the other hand, using equations (S4a) and (S3), we can obtain

$$\frac{\partial\psi_{\mathrm{S}}}{\partial\phi} = \frac{s_0\cos\delta_s}{s_0^2 - s_3^2}\left(\tilde{A}_x\frac{\partial\tilde{A}_y}{\partial\phi} - \tilde{A}_y\frac{\partial\tilde{A}_x}{\partial\phi}\right) - \frac{s_1 s_3}{2(s_0^2 - s_3^2)}\left(\frac{\partial\delta_y}{\partial\phi} - \frac{\partial\delta_x}{\partial\phi}\right). \tag{S14}$$

With relation of $s_0\left(\frac{\partial\delta_x}{\partial\phi} + \frac{\partial\delta_y}{\partial\phi}\right) - s_1\left(\frac{\partial\delta_y}{\partial\phi} - \frac{\partial\delta_x}{\partial\phi}\right) = 2\left(\tilde{A}_x^2\frac{\partial\delta_x}{\partial\phi} + \tilde{A}_y^2\frac{\partial\delta_y}{\partial\phi}\right)$ and equations (S13a), (S13b), and (S14), the following equations are obtained

$$s_0\frac{\partial\psi_{\mathrm{PR}}}{\partial\phi} = -(s_0 + s_3)\frac{\partial\psi_{\mathrm{S}}}{\partial\phi} - \left(\tilde{A}_x^2\frac{\partial\delta_x}{\partial\phi} + \tilde{A}_y^2\frac{\partial\delta_y}{\partial\phi}\right), \tag{S15a}$$

$$s_0\frac{\partial\psi_{\mathrm{PL}}}{\partial\phi} = (s_0 - s_3)\frac{\partial\psi_{\mathrm{S}}}{\partial\phi} - \left(\tilde{A}_x^2\frac{\partial\delta_x}{\partial\phi} + \tilde{A}_y^2\frac{\partial\delta_y}{\partial\phi}\right). \tag{S15b}$$

Through further derivation with equations (S15a) and (S15b), we can finally obtain

$$\tilde{A}_x^2\frac{\partial\delta_x}{\partial\phi} + \tilde{A}_y^2\frac{\partial\delta_y}{\partial\phi} = -s_0\frac{\partial\psi_{\mathrm{PR(L)}}}{\partial\phi} \mp (s_0 \pm s_3)\frac{\partial\psi_{\mathrm{S}}}{\partial\phi}. \tag{S16}$$

Then, substituting equation (S16) into equation (S9b), we can solve the OAM charge. In equation (S16), the first term is the gradient of spiral spatial phase known as topological Pancharatnam charge, and the second term is the SOP-related charge.

B. OAM charge of vector beams generated by two scalar vortex beams

General vector beam can be generated by mode expansion as [S5]

$$|\Phi_{\mathrm{E}}\rangle = \frac{1}{\sqrt{2}}\cos\left(\frac{\theta}{2}\right)(\hat{x} - i\hat{y})e^{-il_{\mathrm{L}}\phi} + \frac{1}{\sqrt{2}}\sin\left(\frac{\theta}{2}\right)(\hat{x} + i\hat{y})e^{-il_{\mathrm{R}}\phi - i\varphi_0}. \tag{S17}$$

Thus, with equation (S11), we can obtain

$$\frac{\partial \psi_{\mathrm{PR(L)}}}{\partial \phi} = -l_{\mathrm{R(L)}}. \tag{S18}$$

Further, the Stokes parameters can be deduced

$$\begin{aligned} s_1 &= \sin\theta \cos\left(-\left(l_{\mathrm{L}} - l_{\mathrm{R}}\right)\phi - \varphi_0\right), \\ s_2 &= \sin\theta \sin\left(-\left(l_{\mathrm{L}} - l_{\mathrm{R}}\right)\phi - \varphi_0\right). \end{aligned} \tag{S19}$$

Thus, with equations (S4a) and (S3), we obtain

$$\psi_{\mathrm{S}} = -\left(\left(l_{\mathrm{L}} - l_{\mathrm{R}}\right)\phi + \varphi_0\right)/2. \tag{S20}$$

Then,

$$\frac{\partial \psi_{\mathrm{S}}}{\partial \phi} = -\left(l_{\mathrm{L}} - l_{\mathrm{R}}\right)/2. \tag{S21}$$

Substituting equations (S18) and (S21) into equations (S9b) and (S16), the OAM charge can be written as

$$l = l_{\mathrm{R}} + \frac{\left(l_{\mathrm{L}} - l_{\mathrm{R}}\right)}{2}\left(1 + \sin 2\chi_{\mathrm{S}}\right) = l_{\mathrm{TPC}}^{\mathrm{R}} + \frac{\left(l_{\mathrm{L}} - l_{\mathrm{R}}\right)}{2}\frac{\Omega_{\mathrm{R}}}{2\pi} = l_{\mathrm{TPC}}^{\mathrm{R}} + \frac{\phi_{\mathrm{gO}}^{\mathrm{R}}}{\pi}, \tag{S22a}$$

$$l = l_{\mathrm{L}} - \frac{\left(l_{\mathrm{L}} - l_{\mathrm{R}}\right)}{2}\left(1 - \sin 2\chi_{\mathrm{S}}\right) = l_{\mathrm{TPC}}^{\mathrm{L}} - \frac{\left(l_{\mathrm{L}} - l_{\mathrm{R}}\right)}{2}\frac{\Omega_{\mathrm{L}}}{2\pi} = l_{\mathrm{TPC}}^{\mathrm{L}} - \frac{\phi_{\mathrm{gO}}^{\mathrm{L}}}{\pi}. \tag{S22b}$$

where $l_{\mathrm{TPC}}^{\mathrm{R(L)}} = l_{\mathrm{R(L)}}$ is topological Pancharatnam charge that is referenced to right (left) circularly polarized field, $\Omega_{\mathrm{R(L)}}$ is the solid angle formed by the swept surface area of SOP revolving around the south (north) pole on the Poincaré sphere, and the $\phi_{\mathrm{gO}}^{\mathrm{R(L)}}$ is the equivalent SOP-related phase induced by space-variant SOP.

C. Reference field with general SOP

In the main text, the reference field was selected as right (left) circularly polarized field in order to induce an elegant expression (see equation (7) in the main text) for calculating the OAM charge. But it does not mean that choice of general reference fields would lead to any deviation. Here, we provide the calculation process with simulation for the case of using general reference field. For easy calculation, the selected reference field $|\Phi_{\mathrm{A}}\rangle$ is located on the $s_2 - s_3$ plane, and rotates an angle of α relative to $|\Phi_{\mathrm{L}}\rangle$ around s_1 axis. Figures S1(a) and S1(b) illustrate coordinate systems with the reference field of $|\Phi_{\mathrm{L}}\rangle$ and $|\Phi_{\mathrm{A}}\rangle$, respectively. In Fig. S1(a), the contracted 3D coordinate (red axes of $x - y - z$) holding $|\Phi_{\mathrm{L}}\rangle$ located at z direction coincide with coordinate of the Poincaré sphere. Thus the investigated vectorial vortex field $|\Phi_{\mathrm{E}}\rangle$ in this coordinate can be expressed as $(2\psi_{\mathrm{S}}, 2\chi_{\mathrm{S}})$. After coordinate transformation with assuming the new reference $|\Phi_{\mathrm{A}}\rangle$ located at z' direction, as shown in Fig. S1(b), $|\Phi_{\mathrm{E}}\rangle$ in the new contracted 3D coordinate (magenta axes of $x' - y' - z'$) can be expressed as $(2\psi'_{\mathrm{S}}, 2\chi'_{\mathrm{S}})$.

Firstly, we consider the calculation of OAM charge with reference $|\Phi_{\mathrm{L}}\rangle$, combining equations (S18), (S21), (S22b), and (S9b), we rewrite the OAM charge with the reference field $|\Phi_{\mathrm{L}}\rangle$, it reads

$$l = \frac{\iint I_E \left(-\frac{\partial \psi_{PL}}{\partial \phi} + \frac{\partial \psi_S}{\partial \phi} \frac{\Omega_L}{2\pi} \right) r dr d\phi}{\iint I_E s_0 r dr d\phi}. \tag{S23}$$

In equation (S23), there are three parts in numerator: azimuthal gradient of Pancharatnam phase ($-\partial\psi_{PL}/\partial\phi$), azimuthal gradient of SOP-related phase ($\partial\psi_S/\partial\phi$), and the relevant solid angle ($\Omega_L/2\pi$), which is formed by the swept surface area of SOP revolving around the $|\Phi_L\rangle$ on the Poincaré sphere.

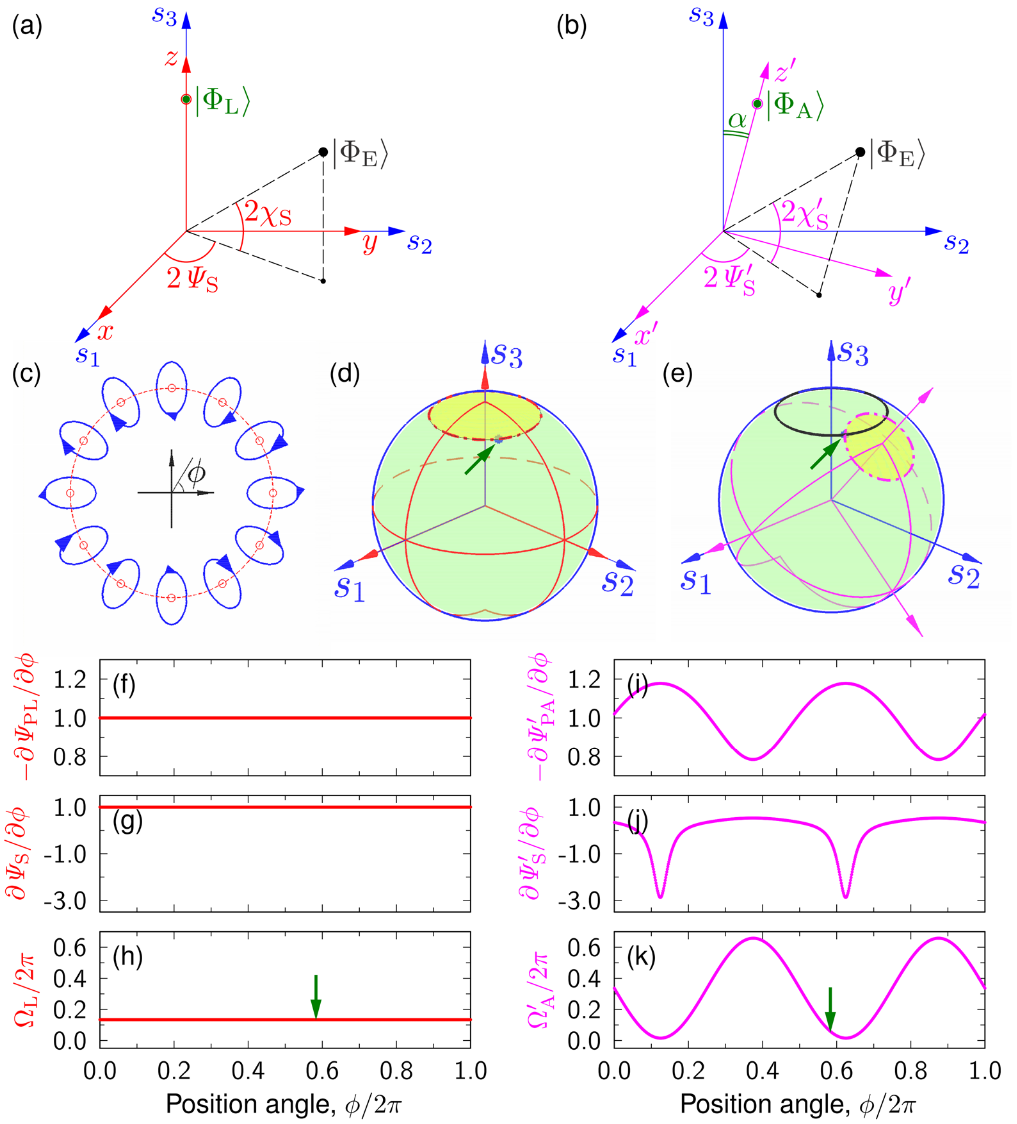

Figure S1. (a) Coordinate of the Poincaré sphere (blue axes) is represented by $s_1 - s_2 - s_3$. Coordinate (red axes), represented by $x - y - z$ and used in the calculations, is contracted with reference field $|\Phi_L\rangle$ located at z direction. Any studied field $|\Phi_E\rangle$ can be expressed as $(2\psi_S, 2\chi_S)$ in the coordinate of $x - y - z$. (b) Rotated coordinate (magenta axes), represented by $x' - y' - z'$ and used in the calculations, is contracted with reference field $|\Phi_A\rangle$ located at z direction. Any studied field $|\Phi_E\rangle$ can be expressed as $(2\psi_S', 2\chi_S')$. (c) The field is simulated in (d-k). (d) and (e) SOP trace on the Poincaré sphere, the yellow surface area corresponds to solid angle of considered SOP marked by green arrow in calculation with reference $|\Phi_L\rangle$ and $|\Phi_A\rangle$, respectively. (f-h) Calculated three parts in equation (S23). (i-k) Calculated three parts in equation (S24).

With similar method, the calculation equation of OAM charge under reference field $|\Phi_A\rangle$ can be written as

$$l = \frac{\iint I_E \left(-\frac{\partial \psi_{PA}}{\partial \phi} + \frac{\partial \psi'_S}{\partial \phi} \frac{\Omega'_A}{2\pi} \right) r dr d\phi}{\iint I_E s_0 r dr d\phi}. \tag{S24}$$

where $\psi_{PA} = \langle \Phi_A | \Phi_E \rangle$ is Pancharatnam phase referenced to $|\Phi_A\rangle$, ψ'_S is the angle under new coordinate (see Fig. S1(b)), and Ω'_A is solid angle of SOP swept area around new reference $|\Phi_A\rangle$.

In order to verify the correctness of the equation (S24), simulations are carried out. Here, we consider the field shown in Fig. S1(c) (the same field shown in Fig.1(a) in main text), and calculate the OAM charge with reference fields of $|\Phi_L\rangle$ and $|\Phi_A\rangle$, respectively. Figures S1(f-h) show the calculated three parts in equation (S23) with reference $|\Phi_L\rangle$, while Figs. S1(i-k) show the calculated three parts in equation (S24) with reference $|\Phi_A\rangle$. In particular, the surface areas of solid angle are illustrated Figs. S1(d, e) for a typical SOP, which is also marked in Figs. S1(d, e, h, k) with green arrows. Although each of three parts is different, the calculated OAM charges of the light beam are equal with two reference fields.

From the above simulations, we believe that OAM charge can be calculated by adopting any reference fields. However, with reference field of right or left circular polarization, an elegant form (equation (7) in main text) could be obtained. Moreover, right or left circular polarization is the natural choice to measure Stokes parameters. Thus, the reference field of right or left circular polarization is adopted in the main text.

References

[S1] J. P. Torres and L. Torner, *Twisted Photons: Applications of Light with Orbital Angular Momentum*. (John Wiley & Sons, 2011).

[S2] M. Born and E. Wolf, *Principles of optics: electromagnetic theory of propagation, interference and diffraction of light*. (CUP Archive, 1999).

[S3] L. Allen and M. J. Padgett, Optics Communications **184**, 67 (2000).

[S4] M. V. Berry, Journal of Modern Optics **34**, 1401 (1987).

[S5] C. Maurer, A. Jesacher, S. Fürhapter, S. Bernet and M. Ritsch-Marte, New Journal of Physics **9**, 78 (2007).
